# A Hydrothermal-Assisted Ball Milling Approach for Scalable Production of High-Quality Functionalized MoS_2_ Nanosheets for Polymer Nanocomposites

**DOI:** 10.3390/nano9101400

**Published:** 2019-10-01

**Authors:** Mojtaba Ahmadi, Omid Zabihi, Quanxiang Li, Seyed Mousa Fakhrhoseini, Minoo Naebe

**Affiliations:** Institute for Frontier Materials, Deakin University, Geelong, Victoria 3216, Australia; ahmadim@deakin.edu.au (M.A.); Omid.zabihi@deakin.edu.au (O.Z.); quanxiang.li@deakin.edu.au (Q.L.); sobhan.fakhrhoseini@deakin.edu.au (S.M.F.)

**Keywords:** two-dimensional nanomaterials, molybdenum disulfide nanosheets, functionalization, hydrothermal process, ball milling, polymer nanocomposites

## Abstract

The most known analogue of graphene, molybdenum disulfide (MoS_2_) nanosheet, has recently captured great interest because it can present properties beyond graphene in several high technological applications. Nonetheless, the lack of a feasible, sustainable, and scalable approach, in which synthesizing and functionalization of 2H-MoS_2_ nanosheets occur simultaneously, is still a challenge. Herein, a hydrothermal treatment has been utilised to reduce the effect of breaking mechanisms on the lateral size of produced nanosheets during the ball milling process. It was demonstrated that the hydrothermal pre-treatment led to the initial intercalation of an organic molecule such as 4,4′-diaminodiphenyl sulfone (DDS) within the stacked MoS_2_ sheets. Such a phenomenon can promote the horizontal shear forces and cause sliding and peeling mechanisms to be the dominated ones during low energy ball milling. Such combined methods can result in the production of 2H functionalized MoS_2_ nanosheets. The resultant few layers showed an average lateral dimension of more than 640 nm with the thickness as low as ~6 nm and a surface area as high as ~121.8 m^2^/g. These features of the synthesised MoS_2_ nanosheets, alongside their functional groups, can result in fully harnessing the reinforcing potential of MoS_2_ nanosheets for improvement of mechanical properties in different types of polymeric matrices.

## 1. Introduction

The first successful isolation of graphene in 2004 [1] has undoubtedly been a motivation for synthesising other types of two-dimensional (2D) materials, capable of showing tunable electrical properties beyond the potential of gapless (zero energy gap) graphene in the next-generation electronics, nanocomposites, and photonic applications [2]. The 2D transition metal dichalcogenides (TMDs) are considered as multi-functional materials because of their excellent electronic, optical, mechanical, and magnetic properties [3]. Molybdenum disulfide (MoS_2_) has been used extensively in researches because of its availability, low cost, and lightweight [4]. Several research groups in the field of photovoltaic, energy storage, and electronic applications have investigated the MoS_2_ potential. This is mainly encouraged by the reduction of bulk MoS_2_ to the monolayer, resulting in the transformation of bandgap from indirect to direct (~2 eV) which is suitable for the targeted electronic applications [5]. Additionally, the MoS_2_ nanosheet shows more ductility compared to the graphene [6] with the yield strength as high as ~23 GPa and Young’s modulus of ~300 GPa [7,8].

The two methods including top-down and bottom-up have been used for synthesising MoS_2_ nanosheets. Methods such as micromechanical exfoliation using scotch tape [9], liquid exfoliation [10], fluid dynamic exfoliation [11], thermal ablation by lasers [12], electrochemical exfoliation method [13], and low-energy ball milling and sonication [14] are some of examples of top-down approaches. On the other hand, methods such as chemical vapour deposition [15], Van der Waal epitaxial growth on substrate [16], and hydrothermal approach [17] are some of common bottom-up strategies. These low yield methods are time-consuming and costly. However, top-down methods offer low cost, fast, and simple solutions. They offer the potential of scalable production [18,19,20]. Nonetheless, these methods still have several limitations. For example, butyllithium intercalation of functionalized MoS_2_ nanosheets can result in 1T-MoS_2_, which is not photoluminescent [21,22]. Consequently, it loses its semiconductivity because of electron transfer from the butyl group of the butyllithium to the MoS_2_ nanosheets [23]. Although it can be recovered at high temperature (e.g., 300 °C) for several days, the obtained product shows excellent sensitivity to the ambient condition [24,25]. Sophisticated electrochemical control and extra pre-expanding treatment are other consequences of such a method. Furthermore, the corrosive alkaline medium can damage the produced nanosheets [26].

The use of a solvent in ultrasonication can be a disadvantage of ultrasonic cleavage. In addition, this method can damage nanosheets because of high shear energy [27,28]. Additionally, this method is not compatible with all types of solvents [25]. In comparison, water-phase mechanical exfoliation has shown several benefits such as immediate functionalization, controllable size, proper operation, environmental-friendly, and scalable production. Nonetheless, the considerable mismatch between surface energies of water and TMDs calls for surfactants and amphipathic polymers [29]. Generally, the concentrations of MoS_2_ for different methods are relatively low. Therefore, there is still a long way toward finding a feasible scalable production approach.

The low energy ball milling is considered as a scalable, efficient thinning method [14,30]. However, this method leads to the production of the MoS_2_ nanosheets with low lateral dimension [14]. Apart from ball milling, a hydrothermal exfoliation method, in which lithium (Li) is used in the presence of water for interaction/exfoliation of MoS_2_, is a promising method [31]. However, harsh reaction conditions, expensive Li compounds, and the residue of Li in MoS_2_ nanosheets limit the true potential of the hydrothermal process in the production of high-quality MoS_2_ nanosheets. Given these limitations, in this study, we propose a modified hydrothermal technique in which a Li-free modifier agent is used for intercalation within the MoS_2_, followed by the mechanical exfoliation using the ball milling process. The use of the hydrothermal technique is not only to facilitate the ball milling process through pre-intercalation but also providing MoS_2_ nanosheets with reactive-ready functional groups. It is hypothesised that the utilisation of pre-intercalated MoS_2_ can result in nanosheets with large lateral dimensions because the hydrothermal process can probably boost the horizontal shear forces in ball milling instead of breaking mechanisms. Therefore, both the synthesis and functionalization of MoS_2_ nanosheets occurs simultaneously. In other words, a mechano-chemically driven reaction [32,33] has been employed for concurrent exfoliation and functionalization of MoS_2_. Functionalization of MoS_2_ nanosheets using this technique left the 2H structure of MoS_2_ intact, and the functional groups can make them more compatible with different polymeric matrices. With respect to the cost-efficiency of the method used in this study, compared to the sonication [26], which is known as a conventional synthesizing approach, the use of butyllithium in sonication method results in the change of polytype of MoS_2_ into 1T; therefore, further post-modification step such as infrared-laser phase reversion is needed to reach 2H polytype. Additionally, the sonication process resulted in chemically inert MoS_2_ nanosheets, which also needs surface treatment if they are supposed to be added into the polymeric matrices. Consequently, sonication is considered as a three-step labour-intensive procedure to reach functionalized MoS_2_ nanosheets. It is also worthy to mention that the yield of sonication is lower than the synthesised approach used in this study. Consequently, compared to sonication, the synthesized method proposed in this research has higher potential for synthesizing functionalized MoS_2_ nanosheets at scale. Furthermore, the method used in this study led to the production of the reactive reinforcing additives suitable for multi-functional nanocomposites preparation. Therefore, in order to find out the reliability of produced MoS_2_ nanosheets in real world applications, the synthesized MoS_2_ nanosheets were incorporated into the different polymeric matrices including thermoset, thermoplastic, and thermoplastic elastomer to find out their reinforcing effects on mechanical properties.

## 2. Experimental Section

### 2.1. Materials

Bulk MoS_2_ (98% purity, density: 5.06 g/cm^3^, average particle size: 6 µm) and 4,4′-diaminodiphenyl sulfone (97%, melting point of 175–177 °C, DDS) were purchased from Sigma-Aldrich Corporation, Castle Hill, New South Wales, Australia and they were used as received. Acetone (90%, boiling point 101–105 °C) and dimethylformamide (DMF, 99.8%, boiling point 152–154 °C), as solvents, were purchased from Sigma-Aldrich Corporation, Castle Hill, New South Wales, Australia. Unsaturated polyester (PS, AROPOL^®^-1472, Nupol, Melbourne, Victoria, Australia), containing 45 wt.% styrene, polyvinyl alcohol (PVA, molecular weight 89,000–98,000, 99% hydrolysed, Sigma-Aldrich Corporation, Castle Hill, New South Wales, Australia), and thermoplastic polyurethane (TPU, Elastollan^®^ C95A, BASF, Ludwigshafen, Germany) were used as matrices for nanocomposites preparation. Methyl ethyl ketone peroxide (MEKP, Butanox-M5, AkzoNobel, Melbourne, Australia) was used as a catalyst.

### 2.2. Nanosheets Preparation

The intercalation and exfoliation of bulk MoS_2_ were carried out by using hydrothermal autoclave and ball milling. In the first step, acetone solutions consisting of 12 wt.% DDS were prepared. Secondly, 1.5 g of bulk MoS_2_ with different weight ratios of MoS_2_: DDS (1:1, 1:2, 1:4, and 1:8) were mixed with the prepared DDS/acetone solutions, separately. Then, the final solutions were placed into hydrothermal autoclave chambers (120 mL), followed by heating at 120 °C for 6 h. Afterwards, they were cooled down to room temperature and dried to obtain different DDS-intercalated MoS_2_ powders with various aforementioned ratios. Subsequently, an overall weight of 4 g of each those dried powders was placed into ball milling jars, containing 400 g stainless steel balls with the diameter of 25 mm, and ignited in a horizontal ball milling at 150 rpm for various time durations including 6 h, 12 h, and 24 h. During the ball milling process, exfoliation, surface functionalization, and size reduction occurred. Finally, for the characterisation, the ball-milled powder mixtures were firstly dispersed in 1 L acetone via gentle sonication for 5 min, and the resultant solutions were kept idle for 24 h. Subsequently, 90% of the upper section of the solutions containing DDS functionalized MoS_2_ nanosheets (F-MoS_2_ nanosheets) was separated from 10% of the bottom section containing large and unexfoliated bulk MoS_2_. The collected solutions were filtered and washed by acetone to remove any excess unreacted DDS. Then, they were dried at a vacuum oven. Yield measurements concerning initial bulk MoS_2_ were 46.4%, 85.8%, and 93.9% with respect to different ball milling times of 6 h, 12 h, and 24 h, respectively.

### 2.3. Polymer Nanocomposites Fabrication

The applicability of F-MoS_2_, compared to the ball-milled MoS_2_ as well as bulk MoS_2_, was studied through the examination of mechanical reinforcing potential of F-MoS_2_ in three different types of polymeric matrices including PS, PVA, and TPU. For this purpose, 0.5% of all polymeric matrices were composed of fillers. With respect to PS, 0.5% of F-MoS_2_ was initially premixed with PS and sonicated for 30 min to achieve a homogenous solution. Subsequently, the curing agent, 1.5% of MEKP, was added during a mild mechanical stirring. In order to remove any bubbles and voids during the mixing process, the vacuum was used. The samples were precured at room temperature for 24 h followed by a post curing at 60 °C for 2 h. For the PVA samples, PVA was initially dissolved in deionised water (5 wt.%) at 100 °C by magnetic stirrer. Simultaneously, the fillers were dispersed in 5 mL water by bath sonication. These prepared solutions were mixed and mechanically stirred for 1 h. The PVA nanocomposite films were then dried under a fume hood for one day followed by 12 h at 60 °C in a vacuum oven. For the preparation of thermoplastic elastomer nanocomposites, a solution of TPU dissolved in DMF was added to a sonicated MoS_2_/DMF suspension (10 mg/mL) and further sonicated for 30 min in a sonication bath. The TPU/MoS_2_ formed a network, and it was coagulated by pouring ~1 litre cold water into the TPU/MoS_2_ suspension. Once the separation of polymer occurred, the products were dried at 80 °C under vacuum for 48 h. Finally, MoS_2_ reinforced TPU was hot-pressed at 220 °C for 10 min to produce uniform nanocomposite sheets. Similarly, neat samples containing bulk MoS_2_ and ball-milled untreated MoS_2_ were prepared.

### 2.4. Characterisations

Samples were analysed by a Bruker-Vertex Fourier transform infrared (FTIR) (Bruker Ltd., Billerica, MA, United States) spectrometer in the wavenumber range of 4000 cm^−1^ to 600 cm^−1^. X-ray photoelectron spectroscopy (XPS) was conducted on a Kratos AXIS Nova (Kratos Analytical Ltd., Manchester, United Kingdom) with an Al Kα X-ray source, and the relevant data processing was done using CasaXPS software package (Casa Software Ltd. Teignmouth, United Kingdom). Thermal gravimetric analyses (TGA) were conducted using Perkin–Elmer TGA (Ta Instrument, Sydney, New South Wales, Australia) from 30 °C to 800 °C at the heating rate of 10 °C·min^−1^ under nitrogen ambient to evaluate functionalization degree of MoS_2_ nanosheets. A transmission electron microscope (TEM) (JEOL2100 FEGTEM at 200 KV, JEOL Ltd., Tokyo, Japan) was utilised to characterise the morphologies and structures of MoS_2_ nanosheets. A laser diffraction particle size analyser, MASTERSIZER 2000, manufactured by Malvern Panalytical Ltd., Royston, United Kingdom, studied the particle size distribution of MoS_2_ solution. The crystalline structure of produced nanomaterial was investigated by X-ray diffraction (XRD) measurements using PANalytical X’Pert (Malvern Panalytical Ltd., Royston, United Kingdom) Powder Diffractometer (Cu Kα radiation with λ = 1.54184 Å) in the range of 10°–70° (2θ°), operating at 45 kV and 30 mA. Additionally, a scanning electron microscope (SEM) (Hitachi S4500 Zeiss Supra 55VP, ZEISS, Oberkochen, Germany) and atomic-forced microscopy (AFM) (Bruker Multimode 8, Bruker Ltd., Billerica, MA, United States) were employed for further morphology characterisation via the ScanAsyst in Air mode. The Brunauer-Emmett-Teller (BET) surface area was investigated by N_2_ adsorption-desorption at 196 °C utilising a Quantachrome Autosorb instrument (Quantachrome Corporation, Boynton Beach, FL, United States). The nanosheets were analysed with Renishaw inVia Raman microscope (Renishaw, Wotton-under-Edge, United Kingdom) to investigate their chemical structures and the effect of modification. Three spectra from each sample were collected to compare their homogeneity. UV-Vis spectroscopy was also performed by a Cary 300 UV-vis spectrophotometer (Agilent Technologies, Inc., Santa Clara, CA, United States). Water contact angles on prepared sample disks were studied using a KSV Model CAM101 Contact Angle Meter (KSV Instruments Ltd., Helsinki, Finland) equipped with an Olympus DP70 high-resolution microscope. The potential of F-MoS_2_ in the improvement of tensile properties for all samples comprising of PS, PVA, and TPU samples were studied by using an Instron Universal machine (Instron Pty Ltd., Melbourne, Victoria, Australia) according to ASTM D638 for PS and ASTM D822 for both PVA and TPU samples.

## 3. Results and Discussion

### 3.1. Morphological Characteristics

**Figure 1:**
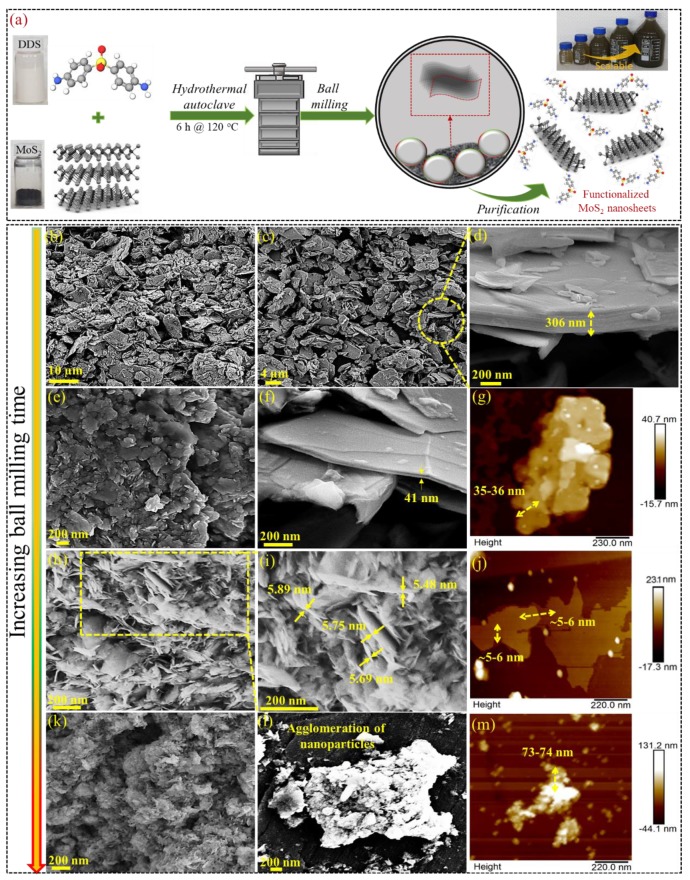
(**a**) The schematic presentation of F-MoS_2_ nanosheets preparation; SEM and AFM images of (**b**–**d**) bulk MoS_2_; (**e**–**m**) F-MoS_2_, for different ball milling duration as indicated in the image.

When it comes to the preparation of the F-MoS_2_ nanosheets, the two steps including hydrothermal treatment and ball milling are of importance since these steps led to both delamination of stacked layers of MoS_2_ as well as surface treatment (Figure 1a), noting that different ball milling times resulted in different nanosheet sizes. In this regard, several microscopic techniques including SEM and AFM methods are employed to study the structures and morphologies of MoS_2_ before and after the exfoliation process (Figure 1b–m). The bulk MoS_2_ shows particles in micrometre size, in which the layers are stacked together with layers thickness as high as 306 nm (Figure 1b–d). However, after ball milling at different durations, the reduction of thickness and lateral dimension occur. When it comes to the ball milling of samples for a short time, e.g., 6 h the reduction of thickness is considerable but not sufficient for producing few-exfoliated layers (Figure 1e,f). Such observation is also confirmed by AFM image (Figure 1g), indicting 35–36 nm thickness. Once ball milling duration increases to 12 h, (Figure 1h,i), the delamination forces are high enough to separate layers considerably. The mechanisms by which the separation of the layers occurs are discussed in the next section. The produced nanosheets have an average thickness of 6 nm comprising 4–5 single nanosheets (Figure 1j) [22,34,35]. A 24 h ball milling, on the other hand, crushes the bulk MoS_2_ down into small fragmentations, which are no longer in the form of nanosheets (Figure 1k). These small fragmentations (less than 100 nm) can result in micron size agglomeration (Figure 1l,m), denoting that higher time can only break nanosheets instead of inducing further mechanical exfoliation.

**Figure 2:**
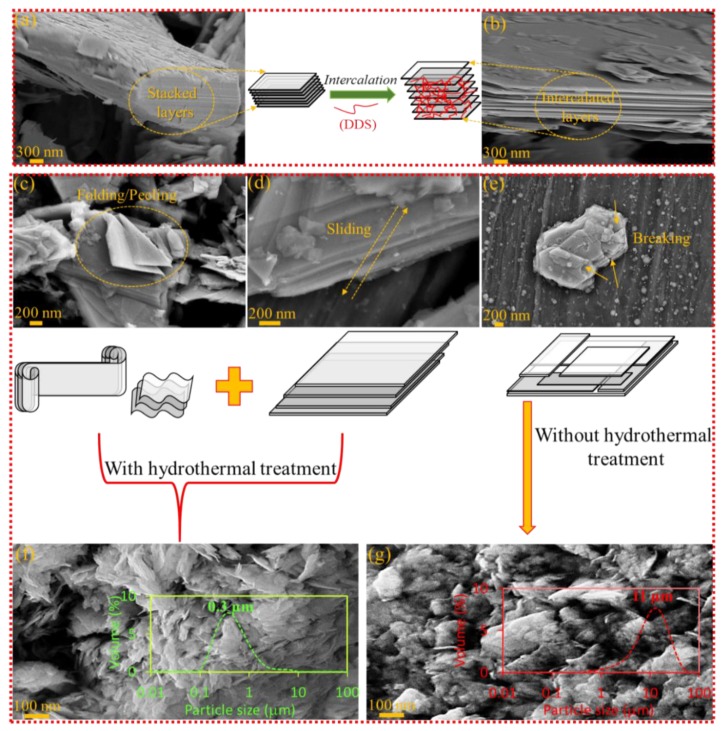
(**a**–**g**) Schematic presentations, SEM images of mechanisms and particle size distribution of hydrothermal and ball milling process in preparation of F-MoS_2_ nanosheets prepared by 12 h milling.

The effect of hydrothermal treatment and ball milling step on F-MoS_2_ nanosheets is presented in Figure 2. Before exposure to high pressure and temperature medium, the stacked structure of bulk MoS_2_ is completely evident indicating that there are not any interlayer gaps between the sheets (Figure 2a). However, the insertion of DDS into these structures is expected to detach bundles of stacked layers from each other, and consequently, some gaps within the stacked layers of DDS-intercalated MoS_2_ can be observed (Figure 2b). It is also clear that the addition of DDS between layers cannot lead to exfoliation and only intercalation is bound to be provided. In general, the ball milling process can result in two main forces including compressive and shear forces [36]. These types of forces can have a folding effect (Figure 2c), sliding effect (Figure 2d), and breaking consequence (Figure 2e) on the bulk MoS_2_. These effects lead to the exfoliation of stacked layers of MoS_2_. The breaking can result in smaller nanosheets holding smaller lateral dimensions. In order to understand the efficiency of hydrothermal treatment, the effect of 12 h ball milling processes of F-MoS_2_ with hydrothermal treatment (Figure 2f) and without hydrothermal treatment (Figure 2g) on the morphology of the resulting F-MoS_2_ nanosheets was studied by SEM. As can be seen, compared to conventional ball milling of F-MoS_2_, nanosheets with larger lateral dimensions can be formed by introducing hydrothermal treatment before ball milling. In this case, it is assumed that folding and peeling mechanisms are dominated. However, smaller sheets, as well as the agglomeration of small nanoparticles, can be seen for F-MoS_2_ samples without hydrothermal step. In such a condition, it is assumed that the breaking mechanism plays a crucial role in nanosheet formation. Therefore, it can be hypothesised that the hydrothermal treatment can improve the horizontal shear forces on the bulk MoS_2_, leading to sliding-peeling effect [37]. In order to prove such deduction, particle size distribution was also measured, and their results are presented in Figure 2f,g. As can be seen, the particle size of the samples treated with hydrothermal is below 1 µm, whereas the smaller nanoparticles arising from breaking mechanisms led to the formation of agglomeration as large as 10 μm. Additionally, the narrow distribution observed for samples without hydrothermal treatment can indicate the formation of fine nanoparticles with small lateral dimensions. Such observation can occur due to breaking mechanisms.

**Figure 3:**
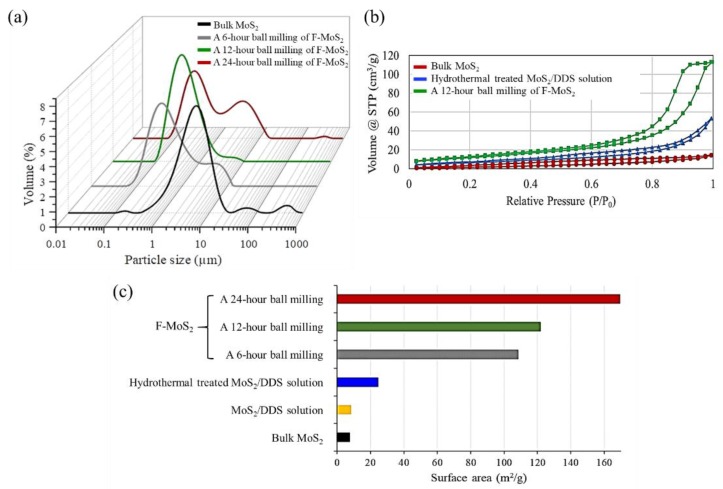
(**a**) Particle size distribution, (**b**,**c**) N_2_ adsorption-desorption isotherms and calculated BET surface area.

Dynamic light scattering measurement is another evaluation for studying the effect of ball milling time on the size of nanosheets in different samples. Figure 3a shows the plot of the distribution of measured sheet sizes for different samples. Although mechanical stirring and sonication are parts of dynamic light scattering experiments, bulk MoS_2_ tends to form agglomerations in the solution. Apart from the main peak for bulk MoS_2_ showing particle size lower than 10 µm, these agglomerations can result in multiple peaks higher than 100 µm. However, after ball milling for different durations, the peaks shift to lower particle size areas. When the ball milling time is short (6 h), a new peak less than 1 µm appears indicating the reduction of size. Nonetheless, since the ball milling time is not enough, still some large particles, higher than 10 µm, are observed. Once the duration of ball milling increases to 12 h, most of the MoS_2_ is converted into the particles with a size of less than 1 µm, having a narrow distribution. Such results are in agreement with our AFM and SEM observations. However, although the ultimate ball milling time (24 h) can further decrease the particle size, the smaller particles tend to agglomerate. These agglomerations can result in the appearance of a peak of around 10 µm.

As discussed previously, when the bulk MoS_2_ is exfoliated into a few layers, the size and thickness decrease, which can affect the surface area. As presented in Figure 3b, using N_2_ adsorption-desorption isotherms, BET surface area of bulk and exfoliated MoS_2_ nanosheets were investigated. The BET surface area of bulk MoS_2_, hydrothermally treated MoS_2_, 12 h ball-milled MoS_2_ were calculated and reported in Figure 3c. As illustrated, the BET surface area of bulk MoS_2_ is ~7.4 m^2^/g. The use of DDS/acetone without hydrothermal process does not affect the surface area considerably. However, after hydrothermal treatment, an increase from 7.4 m^2^/g to 24.3 m^2^/g is seen which can be due to the intercalation of DDS within bulk MoS_2_ layers. Additionally, the ball-milling process increases surface area. The maximum surface area is attributed to 24 h ball milling, where its value as high as 169.3 m^2^/g. Compared to 24 h ball milling, lower values of surface area, i.e., 121.8 m^2^/g and 108.2 m^2^/g are seen for 12 h and 6 h ball-milling processes, respectively. As a result, it has been shown that the ball milling process can increase the surface area of F-MoS_2_ nanosheets [33]. The higher surface area can result from the formation of small nanoparticles, which have more edges to expose. These small nanoparticles would have a higher tendency to form agglomerations. In such condition, the repulsive forces are not strong enough to stabilise the solution; therefore, the appearance of the peaks at a larger size in the results of the particle size distribution can be expected.

**Figure 4:**
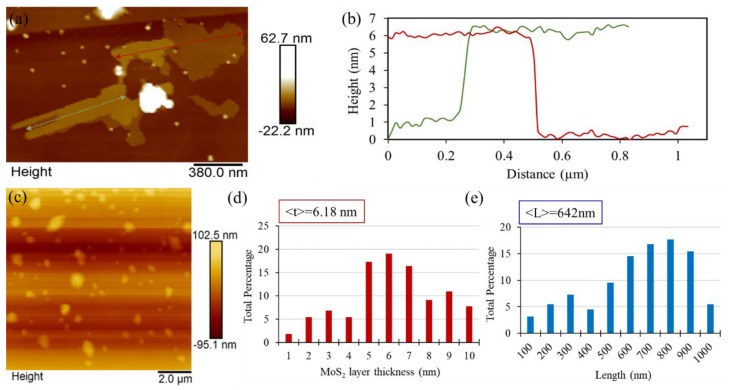
(**a**) An AFM image, (**b**) thickness profile, and (**c**–**e**) AFM image and its histograms of a 12 h ball milling of F-MoS_2_ nanosheets.

As a representative, Figure 4a,b demonstrate AFM image and thickness profile of F-MoS_2_ nanosheets after 12 h ball milling, showing a lateral dimension as large as ~400–800 nm (at least in one direction) and a thickness about 6 nm. Additionally, AFM observations were performed on average 220 nanosheets in different scanning areas with the scan size of ~10 µm × 10 µm (Figure 4c). The statistical analyses on AFM observations show that the samples exposed to a 12 h ball milling have an average thickness of ~6.18 nm and lateral dimension ~642 nm (Figure 4d,e). Additionally, around 64.5% of nanosheets holds the lateral dimension between 600 to 900 nm, confirming a fair distribution of nanosheet size.

### 3.2. Chemical Structure of F-MoS_2_ Nanosheets

**Figure 5:**
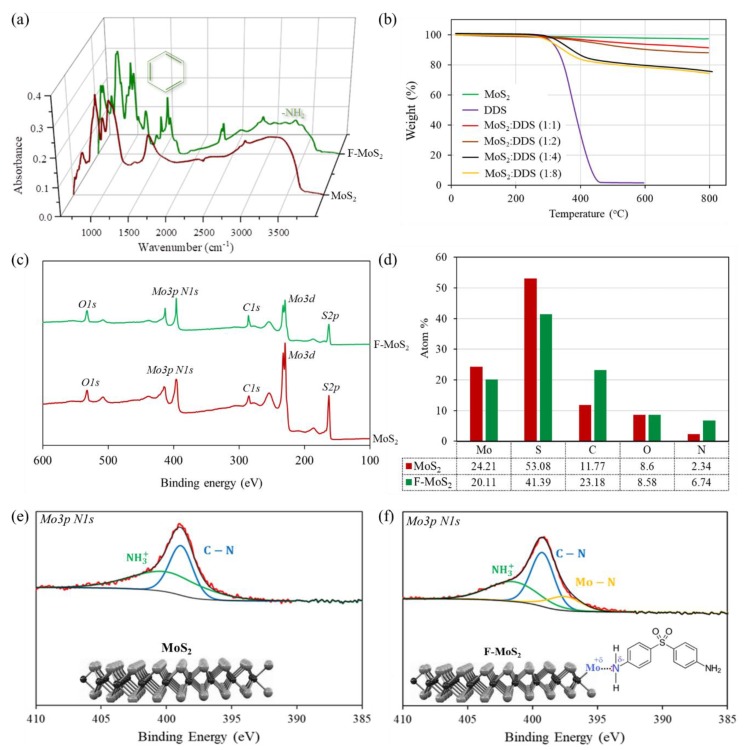
(**a**) FTIR, (**b**) TGA, and (**c**–**f**) XPS analyses of both bulk MoS_2_ and F-MoS_2_.

Different characterisations were used to investigate the surface functionalization of MoS_2_ nanosheets with DDS. The FTIR spectra of bulk MoS_2_ and F-MoS_2_ nanosheets are shown in Figure 5a. Considering the bulk MoS_2_, the Mo-S stretching vibration band peak is around 469 cm^−1^ (below 600 cm^−1^) [38] and noticeable peaks are not expected to be seen in wavenumbers ranging from 600 cm^−1^ to 4000 cm^−1^ [39]. Based on this fact, the observed bands in this range could be related to the oxidation state of MoS_2_, or it may arise from the water or gas coming from exposure to the atmosphere [40]. The bands around 818 cm^−1^, 773 cm^−1^, and 675 cm^−1^ are related to the symmetric and asymmetric stretching vibration of Mo-O [41]. The wavenumber of 1149 cm^−1^ is assigned to the asymmetric S=O and S-O stretching vibrations [42]. The bands at 1636 cm^−1^ and 3365 cm^−1^ are assigned to hydroxyl and water on MoS_2_. The peaks at 2340 cm^−1^ and 2361 cm^−1^ appeared because of carbon dioxide to the MoS_2_ surface [43]. The observation of bands around 2900 cm^−1^ such as 2920 cm^−1^ can be associated with bridge vibration of H_2_O-CO_2_ [40]. After modification, the appearance of strong and weak peaks around 1497 cm^−1^, 1589 cm^−1^, 1685 cm^−1^, and 1453 cm^−1^ can originate from absorption of benzene skeleton vibration, which is achieved by the DDS functionalization [44]. The peaks between 3300 cm^−1^ to 3500 cm^−1^ can be attributed to –NH_2_. Such observation can confirm the success of functionalization. To better confirm the success of DDS functionalization, the TGA measurements were also employed and the results are shown in Figure 5b. A minor weight loss (less than 5%) can be seen during the heating from ambient temperature to 800 °C for bulk MoS_2_. As-received bulk MoS_2_ did not show any degradation below 500 °C [45]. In the case of F-MoS_2_, the weight loss over 300 °C is due to the degradation of DDS molecules attached on the MoS_2_ surface. However, the reduction of weight below 300 °C can be associated with the evaporation of the absorbed water [46]. At a low ratio of MoS_2_ to DDS such as ratios of 1:1 and 1:2, low amount of functional groups is attached on nanosheet surface, which can be negligible, whereas at higher ratio including 1:4 and 1:8, the weight loss around 11% can be detected for F-MoS_2_ nanosheets. It is evident that at the highest ratio (1:8), although the amount of DDS was doubled, the amount of grafting was almost similar, indicating that 1:4 ratio can be considered as the optimum ratio in this study (Figure 5b). Additionally, XPS analysis can show the elemental composition of both bulk MoS_2_ and F-MoS_2_, indicating the success of MoS_2_ functionalization (Figure 5c). Furthermore, there is a change in peak intensities and element compositions. The nitrogen content increases in the XPS spectrum of F-MoS_2_ nanosheets confirming the presence of DDS on the MoS_2_ surface (Figure 5d). It is also noted that before any modification, the bulk MoS_2_, shows two distinct chemically nitrogen atoms related to C-N and NH_3_^+^ (Figure 5e) coming from abovementioned contamination in the FTIR analysis. However, after modification, alongside nitrogen types corresponding to C-N and NH_3_^+^ holding energies at 399.3 eV and 401.7 eV, a peak of Mo-N bond at binding energy of 397.2 eV implies that the nitrogen atom of NH_2_ in DDS is attached to the molybdenum atom. This mechanism can happen when the sulfur vacancy is generated by the exfoliation and in-situ functionalization during ball milling [47,48]. Chalcogen atoms (sulfur, S) of MoS_2_ in the basal plane of nanosheets are saturated; therefore, they are not highly reactive, whereas the metal site (molybdenum, Mo) of MoS_2_ can form bond with -NH_2_ of DDS during exfoliation via sulfur vacancy, as schematically shown in Figure 5f [49].

**Figure 6:**
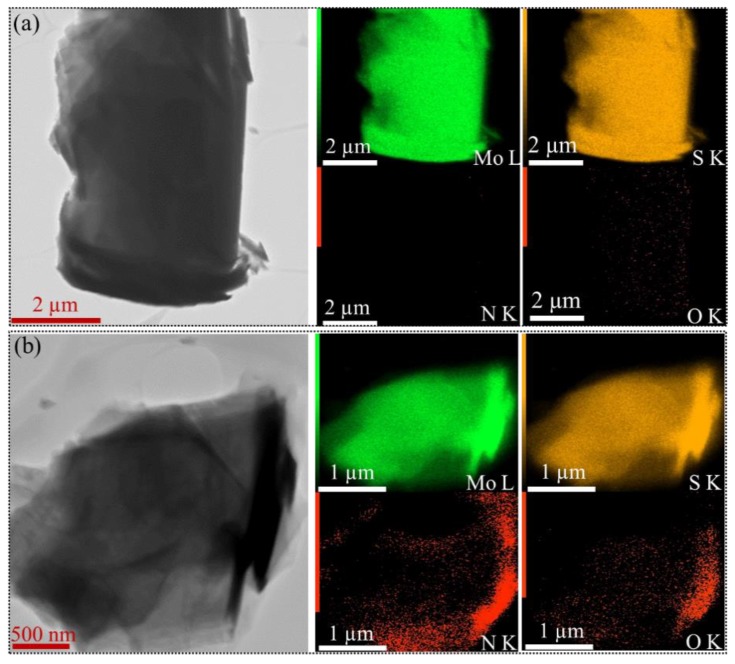
TEM images and elemental mapping for (**a**) bulk MoS_2_ and (**b**) F-MoS_2_ nanosheets.

Such discussion was also confirmed by TEM and elemental mapping done by EDS, Figure 6. It is worthy of mentioning although the nitrogen atom can be detected very well in XPS studies, such observation cannot be detected for bulk MoS_2_. The contradiction between EDS and XPS studies can be related to the non-accurate nature of EDS method for low atomic number elements [50].

### 3.3. Structural Characteristics of F-MoS_2_ Nanosheets

**Figure 7:**
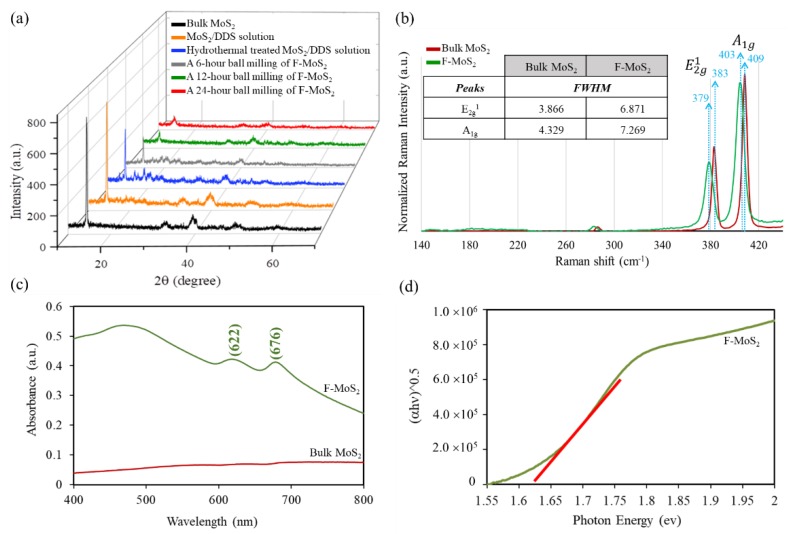
(**a**) XRD patterns, (**b**) Raman spectra and (**c**,**d**) UV-Vis absorption spectra and bandgap calculation of various samples.

Figure 7 shows deeper insight into the structural properties of both bulk MoS_2_ and functionalized MoS_2_ nanosheets. The XRD evaluation for 2θ° ranging from 10° to 70° were measured for as-received bulk MoS_2_ and its different types of its treatment. As can be seen in Figure 7a, the bulk MoS_2_ shows the crystallite nature having some typical peaks located at 14.2°, 32.6°, 39.5°, 44.2°, 49.8°, and 58.3°, relating to the (002), (100), (103), (006), (105), and (110) crystal planes of the 2H-MoS_2_ structure [51]. Such observation is inconsistent with characteristic peaks derived from JCPDS card No. 37-1492. The typical feature of MoS_2_ monolayer is that the diffraction arising from layer-stacking status (002) is expected to disappear [17]. The most intensive (002) peak in the observed pattern of as-received bulk MoS_2_ is positioned at 14.38° attributing to *d*_(002)_ = 6.15 Å of the 2H structure of MoS_2_. The reduction of (002) intensity can suggest the reduction in thickness of bulk MoS_2_ (decrease in the number of layers in each tactoid). Additionally, the widened diffraction peaks show the transformation of bulk MoS_2_ to nanosized sheets [52,53]. To investigate the effect of hydrothermal on structural properties of bulk MoS_2_, the XRD patterns of MoS_2_/DDS/acetone before and after hydrothermal process were obtained (Figure 7a). Although there are weak van der Waals interactions among S-Mo-S layers, the existence of DDS/acetone solution in bulk MoS_2_ without hydrothermal cannot overcome these van der Waals interactions due to lack of enough forces; therefore, the stacked layers keep intact. As can be seen in Figure 7a, the use of DDS/acetone solution without hydrothermal does not change the XRD pattern in comparison with the bulk MoS_2_. In other words, the DDS molecules do not affect the interaction of MoS_2_, and the interlayer spacing remains intact. However, after the use of hydrothermal autoclave, a decrease in (002) peak is seen which can be because the high pressure and temperature in the chamber can make DDS agents intercalated between the layers of MoS_2_. Similarly, it has been shown that lithium cations can intercalate between the layers of MoS_2_ by using hydrothermal intercalation process, which is in agreement with our observation [31]. After ball milling of DDS intercalated MoS_2_ for different durations, the intensities of (002) peak reduce considerably, indicating the formation of a few-layer MoS_2_ [39]. A longer ball milling time does not change (002) peak intensity, indicating that longer duration of ball milling is not needed, whereas a shorter time of ball milling is not efficient for producing enough energy to exfoliate layers as much as possible. It is worthwhile to mention that the presence of (002) peak after exfoliation can probably be associated with a certain degree of restacking which is inevitable during the process of final sample collection [54].

The Raman spectra of bulk MoS_2_ before and after DDS modification are illustrated in Figure 7b. MoS_2_ has four Raman-active modes and two infrared-active modes. The former consists of E_1g_, E_2g_^1^, A_1g_, and E_2g_^2^ and the latter includes A_2u_ and E_1u_. The Raman spectrum of bulk MoS_2_ shows Raman shifts of 287 cm^−1^, 383 cm^−1^, and 409 cm^−1^ for E_1g_, E_2g_^1^, and A_1g_, respectively [55]. The E_2g_^2^ cannot be detected probably because of limited rejection of Rayleigh scattered radiation [56]. Additionally, E_1g_ cannot also be seen because of the selection rules [18]. E_2g_^1^ (~384 cm^−1^) mode of the bulk 2H-MoS_2_ crystal shows a strong in-plane vibrational mode which cannot be observed for single-layer of Li-intercalated MoS_2,_ and this can be related to the existence of metastable octahedral coordination. Nonetheless, in this case of observation of the probable existence of octahedral coordination, an intercalation-phase transformation is expected. Herein, based on the abovementioned discussion, producing exfoliated MoS_2_ nanosheets shows that the final product still retains the trigonal prismatic coordination of bulk MoS_2_ due to the existence of E_2g_^1^ [56,57]. Additionally, the nonexistence of J_1_, J_2_, and J_3_ peaks can confirm the 2H phase of F-MoS_2_ [26]. For the F-MoS_2_ nanosheets, both out-of-plane A_1g_ and in-plane E_2g_^1^ vibrations show a shift while broadening, which may also be affected by the increase in temperature and also in-plane strain [56,58,59]. Nonetheless, Raman frequencies of E_2g_^1^ and A_1g_ peaks can be utilised as a trustworthy aspect regarding identifying the number of ultra-thin MoS_2_ layers, in comparison with the intensities and widths of peaks [56,60]. According to literature [10,14], the peaks located at 379 cm^−1^ and 403 cm^−1^ (holding frequency differences of ~24 cm^−1^) confirm that a few exfoliated layers of MoS_2_ are successfully obtained and such Raman results are consistent with literature [60]. Furthermore, nevertheless, the reduction of peak intensities and enhancement of full width at half maximum (FWHM) calculated by Lorentz functions (the table in Figure 7b) can also confirm that the exfoliated few-layer of MoS_2_ was successfully achieved [11,61]. Sook bang et al. [34] discussed that the line broadening could be associated with a reduced crystallite size and a higher amount of defects. They reported that MoS_2_ nanosheets synthesised by the liquid-based exfoliation approach have a line width in the range of 6–7 cm^−1^. Additionally, the use of a chemical vapour deposition technique for synthesising of MoS_2_ monolayers leads to an FWHM ranging from 3.5 to 6.6 cm^−1^ [15]. Although a higher FWHM range is observed in our study, it is worthy of considering that in this study the simultaneous functionalization and synthesis has occurred.

With respect to MoS_2_ monolayer at the K point, spin–orbit interaction can split the two-fold degenerate valence bands into two bands with spin-up and spin-down characters since MoS_2_ single layer holding space group P-6 m2 has T-symmetry but no inversion symmetry. Based on this fact, two direct excitonic transitions namely A1 and B1 can be allowed at K point [62]. The optical measurements on dispersed both bulk MoS_2_ and F-MoS_2_ nanosheets in water are examined by UV-Vis spectroscopy, and their relevant results are shown in Figure 7c. Considering the visible radiation range, F-MoS_2_ nanosheet shows a much higher light absorption in comparison with the bulk MoS_2_. The F-MoS_2_ dispersion shows two peaks between 600 and 700 nm and broadband located around 450 nm. The two peaks located at 622 nm (1.99 eV) and 676 nm (1.83 eV) can be related to the characteristic A1 and B1 direct excitonic transitions of MoS_2_ with the energy split from the valence band spin-orbital coupling [63]. These peaks can be attributed to the characteristics of well-dispersed 2H-MoS_2_ nanosheets, confirming the indirect-to-direct transition and increase of bandgap in MoS_2_ [31,34]. Furthermore, the corresponding energy band gaps of bulk MoS_2_ and F-MoS_2_ were obtained using Tauc plots (from UV–vis spectra), Figure 7d. The calculated band gap is ~1.63 eV, corresponding to the F-MoS_2_ with the number of layers of 4–5 layers. Such property can be useful for future applications including optoelectronic devices and electronic devices [64].

### 3.4. MoS_2_ Application in Reinforcing Polymers

**Figure 8:**
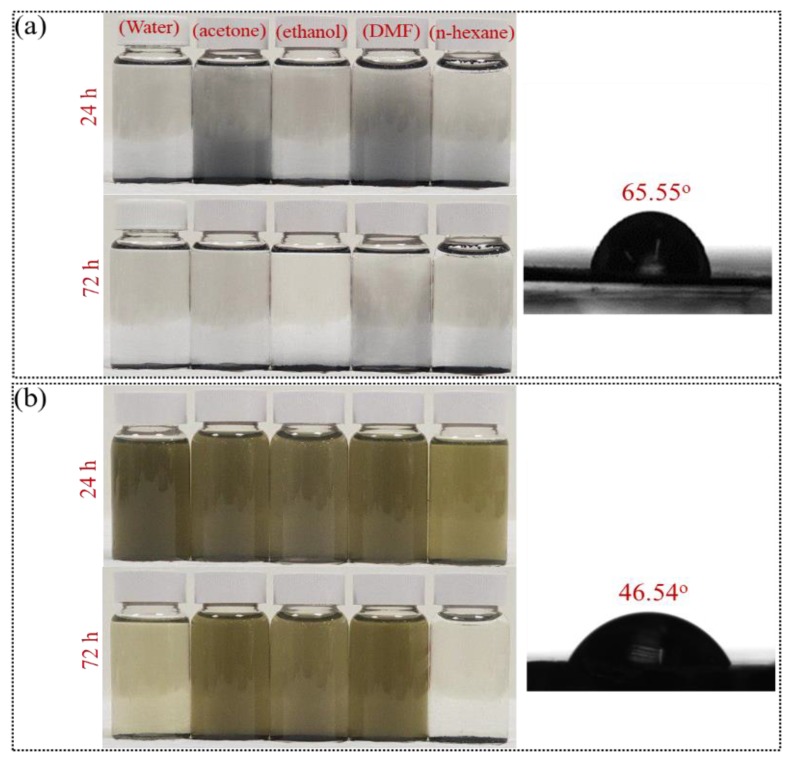
Dispersion/solubility profiles and water contact angle measurements for (**a**) ball-milled MoS_2_ without functionalization and (**b**) F-MoS_2_ nanosheets.

Compared to other applications such as electronics, optical and electrical fields in which synthesising single layers of MoS_2_ is of importance, the possibility of producing multi-functional polymer nanocomposites calls for synthesising surface-functionalized a few-layer MoS_2_ from bulk materials via scalable methods. In this regard, the ball-milling process, as an applicable method for production at scale, can open a new horizon for high-performance nanocomposite developers. In other words, through such process, not only a few-layer MoS_2_ is synthesised but also the surface is equipped with functional groups needed for improvement of interfacial adhesion. To examine such claim, nanocomposites including different types of matrices were prepared. For this purpose, firstly, two main prerequisites, including well dispersion as well as high interfacial interactions, need to be fulfilled to make the best use of reinforcing potential of nanomaterials in polymeric matrices. In this regard, dispersion profiles of different types of MoS_2_ in different types of solvents were of importance. The ball-milled MoS_2_ is an inert material and does not have any specific interactions with different types of solvents. As can be seen in Figure 8a, after a mild sonication for 5 min, the dispersion of MoS_2_ in a vast range of solvents including polar and nonpolar ones are not stable in both 24 and 72 h. However, after functionalization with DDS, alongside the formation of nanosheets, the dispersion levels in different solvents are entirely modified. As illustrated in Figure 8b, water and ethanol, as protic solvents, are capable of hydrogen bonding formation with the amine groups of DDS attached on the surface of MoS_2_ nanosheets [65,66]. Additionally, acetone and dimethylformamide (DMF), as polar aprotic solvents, show the highest of dispersibility since DDS can highly be soluble in such solvents. On the other hand, although n-hexane, a nonpolar solvent, cannot be a suitable solvent for dispersion of DDS the F-MoS_2_ is still stable in n-hexane after 24 h. After 3 days, dispersion of the F-MoS_2_ nanosheets in water is not stable as the initial stage, whereas dipole–dipole interactions with the benzene skeleton of DDS in other solvents including acetone, ethanol, and DMF play pivotal role regarding stability of dispersion for long durations. Such observation can be confirmed by both water contact angle measurement, and the reduction of contact angle from 65.55° to 46.54° can mainly indicate the higher affinity of F-MoS_2_ nanosheets with water because of DDS amine groups.

It is predicted that the stable dispersion, as well as high surface reactivity of F-MoS_2_, make them an excellent reinforcing candidate for polymeric matrices. Considering the results presented in Table 1, compared to neat matrices, the addition of F-MoS_2_ led to an increase in tensile strengths of PS, PVA, and TPU from 27.81 MPa to 36.27 MPa, 24.26 MPa to 27.98 MPa, and 33.16 MPa to 36.69 MPa, respectively. Although functionalization was found to be less effective in terms of enhancement in tensile modulus, higher increases in tensile modulus can be seen for samples containing F-MoS_2_, compared to both bulk and ball-milled MoS_2_. With respect to the strain of samples, the addition of F-MoS_2_ enhanced the strain of PS reaching the value of 0.891%, whereas decreases were seen for PVA and TPU. Such differences can be related to the mechanical instinct behaviour of matrices under tensile loading.

The incorporation of low contents of MoS_2_ nanosheets (≤1 wt.%) has shown an acceptable reinforcing effect for both thermoset and thermoplastic polymers. Since the level of dispersion and the interfacial interactions are important factors for the fabrication of the high-performance nanocomposites, conventional methodologies such as the use of the organic medium, the employment of high shear sonication, and modifier agents have been often suggested by different researchers [67]. In work done by Eksik et al. [68], MoS_2_ nanosheets were prepared by a liquid exfoliation method. They showed that the addition of only 0.3 wt.% of MoS_2_ nanosheets to the thermoset polymer such as epoxy led to an improvement in tensile modulus and tensile strength by ~3% and ~19%, respectively. However, ~25% decrease in tensile strain was seen. This trend can be associated with the agglomeration formations, affecting the interfacial stress transferring. Similar to other 2D nanomaterials, the dissipation of crack energy through different mechanisms such as crack deviation is probably the main mechanism for the enhancement of mechanical properties [69,70]. In other words, as the crack encounters MoS_2_ nanosheets, it can be tilted and twisted resulting in higher energy absorption [68,71]. MoS_2_ nanosheets also have barrier effects, limiting the segmental movement of the polymeric chains. At higher loading of additives, similar to other types of nanomaterials, agglomeration of MoS_2_ and weak interfacial adhesion would have a devastating effect on the composite properties. Furthermore, these formed agglomerations can lead to interference with cross-linking reactions [72,73]. In another research, the surface of MoS_2_ was modified with cetyltrimethylammonium bromide, and the modified nanosheets were added into the epoxy matrix using sonication-assisted solution mixing method using tetrahydrofuran as the solvent. The epoxy matrix reinforced with 0.5 wt.% of treated MoS_2_ showed improvement by ~13% in Young’s modulus, whereas the tensile strength did not change. With respect to tensile strain, 12% decrease in tensile strain was obtained. They mentioned that the incorporation of such treated MoS_2_ nanosheets in matrix made the facture morphology to the rough surface have several dimples, which can be due to the blocking/barrier effect and uniform distribution nanosheets inside polymer matrix [74]. On the other hand, MoS_2_ nanosheets can also strengthen common thermoplastic polymer, including polyethylene oxide, polyethylene, and polypropylene. Similar to thermosetting matrices, solution blending is utilised for the fabrication of thermoplastic nanocomposites. Different contents of MoS_2_ dispersed in solution were mixed with polyethylene oxide solution to produce nanocomposites films. The addition of only 0.9 wt.% MoS_2_ nanosheets led to 53%, 88%, and 73% enhancements of yield stress, Young’s modulus, and elongation at break, correspondingly. The substantial improvement in Young’s modulus indicated the existence of enhanced effective volumes of MoS_2_ nanosheets. As the content of MoS_2_ increased to 0.9 wt.%, the specific spherulite structure and their boundaries became blurred; therefore, in this case, tough fractured surface can be observed, indicating effective stress transferring [75]. Feng et al. [28] showed that the addition of a low amount of MoS_2_ nanosheets (0.7 wt.%) to polyethylene could also enhance the tensile modulus, yield stress and breaking strength by values of 38%, 17%, and 10%, respectively. They showed that MoS_2_ created the roughness to the morphology of fractured surfaces in polyethylene composites because of the pull-out mechanisms. High surface area, uniform exfoliation, defect-free structure, and promoted interactions played key roles in furthering properties such as mechanical properties. The in-situ method is considered as a polymerisation method in which in-situ polymerisation of polypropylene in the presence of Ziegler–Natta catalyst intercalated MoS_2_ nanosheets was carried out to fabricate MoS_2_/polypropylene nanocomposites. It has been shown that the addition of 0.52 wt.% of exfoliated MoS_2_ can result in 11% and 61% improvements in tensile strength and modulus, respectively [76]. Similarly, the in-situ polymerisation of polyethylene was done by using exfoliated-MoS_2_/MgCl_2_-supported Ti-based Ziegler–Natta catalyst. The incorporation of 1.23 wt.% of MoS_2_ nanosheets in the polyethylene resulted in +57%, +114%, and −19% changes in tensile strength, modulus, and elongation at break, respectively [77]. Compared to the aforementioned work, the results reported in this study were considerably better. The addition of 0.5 wt.% of F-MoS_2_ improved that tensile strengths of PS, PVA, and TPU by +30%, +15%, and +11%, respectively, whereas they were only +6%, +8%, and +18% for tensile modulus. Such results are quite comparable with the aforementioned results in literature since the improvement of mechanical properties were obtained by incorporation of only 0.5 wt.% of F-MoS_2_ in this study. Furthermore, the use of F-MoS_2_ had negligible effect on deterioration of tensile strain for both PVA and TPU, whereas the improvement of tensile strain in PS samples was seen. The considerable surface area (121.8 m^2^/g), functional groups attached on the surface of nanosheets, stable dispersion, and few-layer structure of nanosheets can be considered for such achievements in this study.

The strategy used in this study provides a real opportunity for scalable synthesis of functionalized MoS_2_ nanosheets. To compare our developed technique and properties of resulting MoS_2_ nanosheets with what has been reported in the literature, Table 2 is provided. As can be seen, compared to the literature, large lateral dimension, high surface area, and the ease of sample preparation and in-situ functionalization are some of the advantages of the proposed method in this study.

## 4. Conclusions

In this study, a low cost and scalable synthesising method based on ball milling and the hydrothermal process is developed to synthesise and functionalize 2H-MoS_2_ nanosheets. For this purpose, the ball-milling method was combined with hydrothermal treatment in which the DDS was initially intercalated between the stacked layers of bulk MoS_2_. By using such a combined method, the efficiency of ball milling was improved since the sliding and peeling mechanisms occurred instead of breaking mechanism. The produced nanosheets have an average lateral dimension of 640 nm and a thickness of 6 nm with functionalized edges. It was observed that in the absence of hydrothermal treatments, difficulties regarding delamination of stacked sheets of bulk MoS_2_ led to the breakage of nanosheets and reduction of lateral dimensions. Consequently, the nanoparticles are prone to form agglomerations. It was found that using the hydrothermal process and the compression forces had a part in the production of shearing forces. Using the proposed method, large F-MoS_2_ nanosheets having the bandgap as high as 1.63 eV were obtained. Such nanosheets showed enhanced dispersion stability in different solvents. Raman spectra showed that the defects introduced by the process we developed was not high compared to other methods, given the fact that both functionalization and synthesising occurred at the same time with no need for any further processing for the change of polytype. It has been shown that the produced F-MoS_2_ can act as promising reinforcing additives for different types of polymer matrices. In other words, the mechanical performance of polymer nanocomposites showed that the addition of only 0.5 wt.% of F-MoS_2_ led to changes in tensile strengths of PS, PVA, and TPU by +30%, +15%, and +11%, respectively, whereas they were only +12%, +5%, −3% for its counterpart (ball-milled MoS_2_). Similarly, improvements of tensile modulus of PS, PVA, and TPU were around 6%, 8%, and 18%, respectively; however, less improvement was observed for the ball-milled MoS_2_, indicating the effectiveness of functionalization. Compared to the literature, it has been seen that these F-MoS_2_ nanosheets have better performance for composites as long as they have less devastating effect on tensile strain while improving both tensile modulus and strength considerably at low content.

## Figures and Tables

**Table 1:** Tensile properties of PS nanocomposites, PVA nanocomposites, and TPU nanocomposites including different types of MoS_2_.

Sample	Tensile Strength (MPa)	Tensile Modulus (GPa)	Strain (%)
Polyester (PS)			
Neat PS	27.81 ± 1.6	3.75 ± 0.05	0.782 ± 0.021
PS-Bulk-MoS_2_	26.78 ± 2.1	3.53 ± 0.08	0.698 ± 0.042
PS-Ball milled-MoS_2_	31.17 ± 1.5	3.92 ± 0.04	0.865 ± 0.043
PS-F-MoS_2_	36.27 ± 1.8	3.98 ± 0.03	0.891 ± 0.036
Polyvinyl Alcohol (PVA)			
Neat PVA	24.26 ± 2.2	1.81 ± 0.03	83.5 ± 6.9
PVA-Bulk-MoS_2_	22.88 ± 2.8	1.86 ± 0.02	62.3 ± 5.8
PVA-Ball milled-MoS_2_	25.52 ± 2.3	1.91 ± 0.01	85.4 ± 4.3
PVA-F-MoS_2_	27.98 ± 1.8	1.97 ± 0.03	81.9 ± 4.7
Thermoplastic Polyurethane (TPU)			
Neat TPU	33.16 ± 3.5	0.051 ± 0.004	551 ± 8.2
TPU-Bulk-MoS_2_	29.18 ± 2.8	0.054 ± 0.003	402 ± 5.4
TPU-Ball milled-MoS_2_	32.25 ± 3.1	0.057 ± 0.003	545 ± 7.7
TPU-F-MoS_2_	36.69 ± 1.9	0.060 ± 0.002	536 ± 3.9

**Table 2:** Comparison of properties of the produced F-MoS_2_ nanosheets with recent works.

Synthesising Method	Further Processing Step	Lateral Dimension (nm)	Thickness (nm)	Surface Area (m^2^/g)	Comments	Reference
Ball milling	Hydrothermal assisted + DDS	642	6.18	121.8	No need for any solvents during ball milling. Short ball milling time. The existence of reactive groups on edges. Excellent dispersion and high stability. 2H polytype formation.	This study
Ball milling	The use of N-methyl-2-pyrrolidone	100–150	-	20.25	Toxic solvent. 72 h ball milling. 1T polytype formation.	[78]
Ball milling	Sodium cholate	150	2.5–5.3	-	93% Yield. High dispersibility in water without the need for sonication. 2H crystal structure. 6 h ball milling.	[79]
Ball milling	Use of MoO_3_ and sulfur as precursors, followed by calcination at 600 °C for 2 h in argon	<100	<2	-	24 h ball milling. The high density of coordinatively unsaturated surface atoms.	[80]
Ball milling	Use of MoO_3_ and sulfur as precursors, followed by thermal annealing at 350 °C	<100	5.6	61.4	24 h ball milling in argon. Rich exposed edge sites.	[81]
Micromechanical exfoliation using scotch tape	Functionalization with Spherical Gold nanoparticles	-	0.8	-	Differences in the dimension of the nanosheets. Low yield (limitations for scale-up)	[82]
High shear-induced liquid exfoliation	Lithium intercalation by ultrasonication in water	300–800	1–1.2	-	Difficulties in separation, vulnerable to defects, the change of polytype to 1T, and the need for high annealing temperature	[22]
Thermal ablation by lasers	The use of tape followed by laser-thinned	200	0.9	-	The need for the substrate. The limitation of scalable production. Low production rate. Harsh conditions, such as high temperature. Costly procedure.	[12]
Chemical vapour deposition	Using Mo(CO)_6_ and H_2_S precursors on several different substrates, including SiO_2_, sapphire, and amorphous alumina	100	5–20	-	The use of precursors, mostly expensive catalyst, the need for substrate, low yield, and high temperature	[83]
